# Case Report: Microsurgical clipping of a multilobular fenestrated anterior communicating artery aneurysm: technical challenges with video analysis

**DOI:** 10.3389/fsurg.2025.1673454

**Published:** 2025-09-25

**Authors:** Ufuk Erginoglu, Zeynep Arzum Uyaniker, Cagdas Ataoglu, Umid Sulaimanov, Bekir Can Kendirlioglu, Huseyin Erdem Ak, Miner Ross, Mustafa Kemal Baskaya

**Affiliations:** Department of Neurological Surgery, School of Medicine and Public Health, University of Wisconsin-Madison, Madison, WI, United States

**Keywords:** aneurysm clipping, anterior communicating artery (AComA) aneurysm, duplicated/fenestrated aneurysm, duplication, fenestration, multilobular aneurysm, pterional craniotomy

## Abstract

**Background:**

The anterior communicating artery (AComA) is a common site for intracranial aneurysms due to its complex vascular architecture. Fenestrations in the AComA, observed in 7.5%–40% of cases, can disrupt normal blood flow, which increases turbulence and shear stress and heightens the risk of aneurysm formation.

**Case description:**

We report the case of a 67-year-old female who presented with headaches. Imaging revealing a 10 mm unruptured multilobular aneurysm arising from the AComA. Microsurgical clipping was performed via a right pterional craniotomy. The fenestrated nature of the AComA only became apparent after intraoperative full dissection of the AComA complex. Intraoperative Doppler ultrasound and indocyanine green angiography were used to evaluate the vascular anatomy, which was followed by the successful clipping of the aneurysm. Postoperative angiography verified complete obliteration, and the patient recovered without neurological deficit.

**Conclusion:**

The complexity of the AComA anatomy in this case prompted reflection on the limitations of preoperative imaging and the need for careful intraoperative planning. Although advanced imaging modalities, particularly three-dimensional rotational angiography, are crucial for detecting vascular anomalies, ultra-thin bridging vessels may remain undetected, as occurred in this case. Intraoperative recognition of these structures required real-time adaptation to ensure safe dissection and successful aneurysm clipping To our knowledge, this is the first operative video documenting microsurgical clipping of an unruptured aneurysm within a fenestrated AComA. This case underscores the importance of anticipating anatomical variations and adapting surgical strategies to optimize outcomes in complex cerebrovascular procedures.

## Introduction

The anterior communicating artery (AComA), a crucial element of the Circle of Willis (CoW), is the most common site for intracranial aneurysm formation, accounting for approximately 30%–37% of cases (1–5). AComA aneurysms present considerable clinical challenges due to the complex angioarchitecture of the AComA and its proximity to critical neuroanatomical structures. Since AComA aneurysms are situated near pathways integral to visual, cognitive, and endocrine functions, including the optic apparatus, hypothalamus and basal forebrain, these aneurysm carry a heightened risk of morbidity and mortality if ruptured (2, 3, 6–9).

The diverse morphology of AComA aneurysms compounds the complexity of their surgical management. Although saccular aneurysms are the most frequent, AComA aneurysms can also present as multilobular, elongated, or sessile structures (1, 3, 4). Additionally, secondary lobulations or “daughter aneurysms” often appear, adding further difficulty in surgical intervention and contributing to high postoperative morbidity, particularly in ruptured cases (4, 10, 11). Most AComA aneurysms measure approximately 8 × 12 mm. While larger and/or complex aneurysms already pose substantial surgical challenges, the presence of vascular anomalies such as fenestrations intensify these challenges, and significantly increase the complexity and risks associated with surgical intervention (7, 9, 12, 13).

The embryological development of the AComA plays a central role in its anatomical variability. The artery originates as a multi-channeled, plexiform structure by Day 35 of gestation, which typically coalesces into a single vessel between Days 21 and 24. However, incomplete fusion during development can lead to structural variations, including fenestrations and duplications, which often are described interchangeably in the literature due to their similar embryological origins. Autopsy studies report that the incidence of these anomalies ranges from 7.5% to 40%, underscoring the complexity frequently encountered in this region (14–16). These variations are often challenging to distinguish due to the short length of the AComA. These variations also disrupt normal hemodynamics thereby predisposing the vessel to aneurysm formation (14, 15, 17).

Hemodynamic factors play a critical role in aneurysm formation within the AComA, particularly in cases with anatomical variations. In asymmetrical AComA models, stagnation points frequently form at the AComA-A2 junction alongside increased wall shear stress, a factor closely associated with aneurysm formation. As blood flow bifurcates, shear stress along the AComA wall sharply escalates, especially when the AComA lacks adaptive capacity (6, 18, 19). Structural anomalies such as fenestrations further disrupt normal blood flow, elevating turbulence and wall shear stress at arterial junctions, thereby increasing susceptibility to aneurysm formation. Additional variations, such as hypoplasia of the A1 segment, amplify these hemodynamic stressors, particularly at bifurcation points, also create conditions conducive to aneurysm development (6, 14, 19, 20).

From a clinical perspective, these anatomical variations complicate both the diagnosis and management of AComA aneurysms. Advanced imaging modalities, such as three-dimensional rotational angiography (3DRA), are essential for preoperatively identifying these anomalies and for optimal surgical planning. However, even high-resolution imaging may struggle to detect ultra-small bridging arteries within fenestrations, which may be thinner than 0.3 mm (17, 20, 21). Additionally, when an aneurysm sac obscures the presence of an anatomical variation, it leads to unexpected challenges in surgical exposure and aneurysm ligation (4, 11). Thus, thorough preoperative and intraoperative assessment is essential for managing the complex anatomical and hemodynamic demands associated with AComA aneurysms (4).

**Figure 1:**
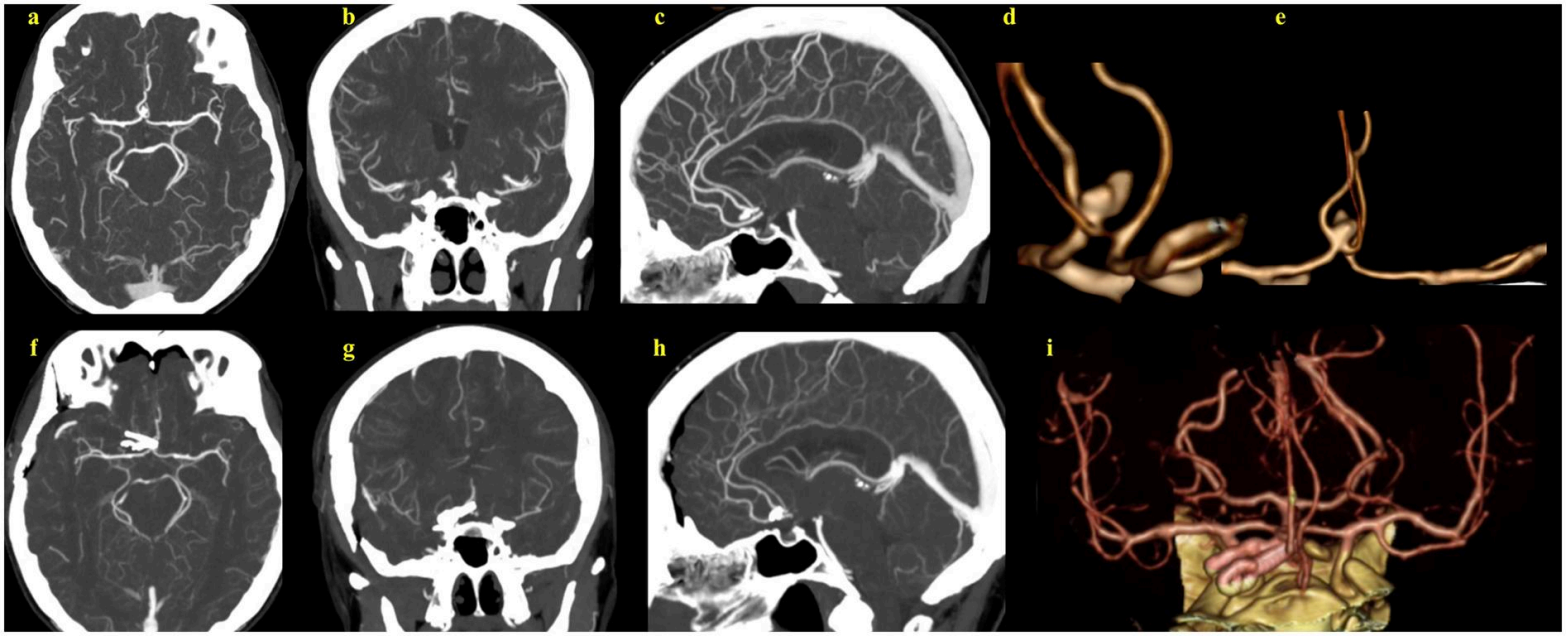
(**a–c**) Preoperative CT angiograms of the complex multilobular anterior communicating artery aneurysm. (**d**,**e**) 2D reconstructions of the preoperative CT angiographic images, providing enhanced aneurysm visualization and its relation to adjacent vascular structures. (**f–h**) Postoperative CT angiograms show successful aneurysm clipping. (**i**) 2D reconstruction of the postoperative images.

**Figure 2:**
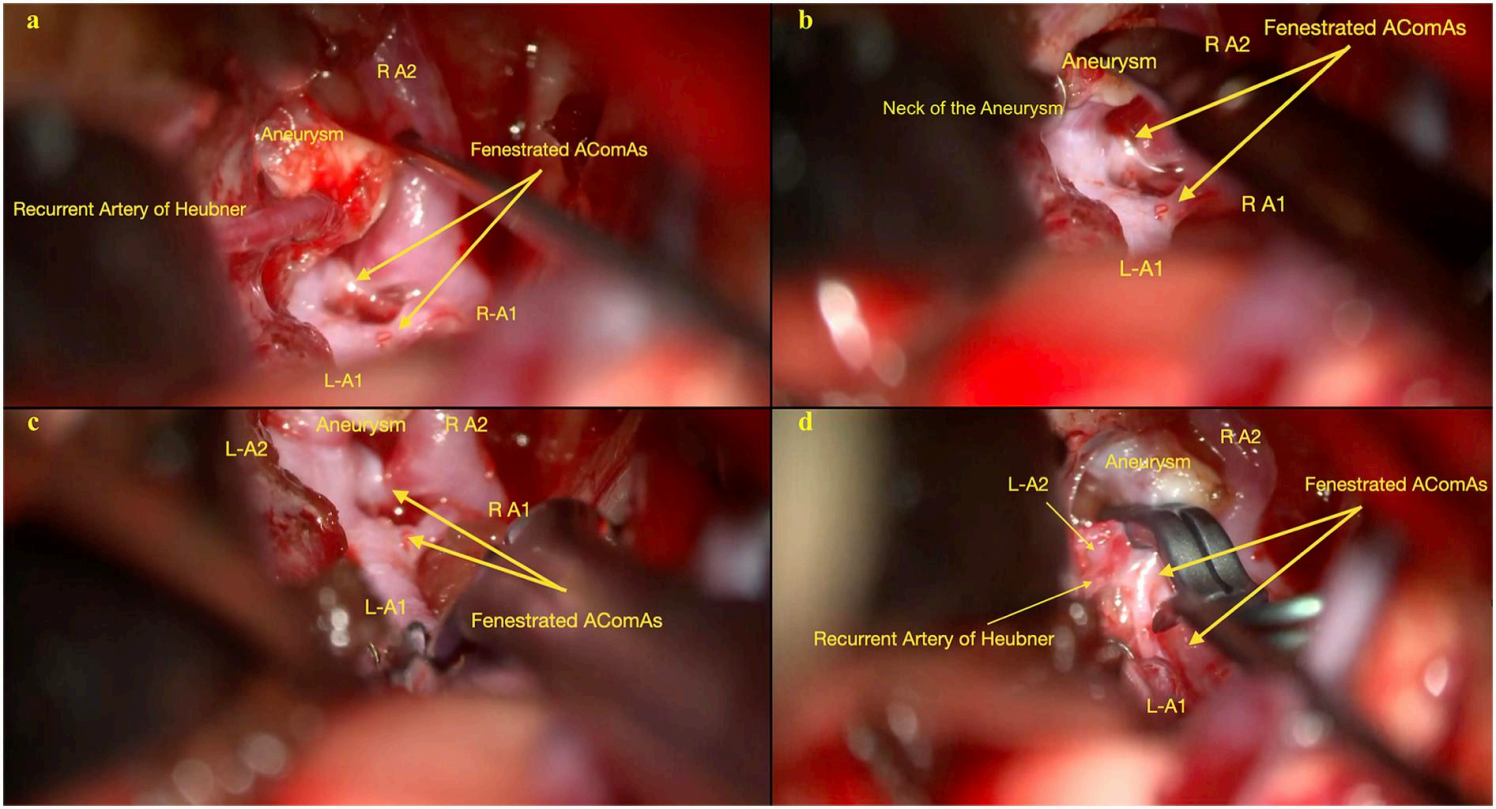
(**a**) Initial depiction of the AComA complex, including the bilateral A1 and right A2 segments, the recurrent artery of heubner, the aneurysm, and the fenestrated AComA. Image shows the attachment of the Heubner artery to the aneurysm dome. (**b**) Another view illustrates the aneurysm neck originating from the larger, more posterior anterior communicating artery and its anatomical relationship with other arteries in the AComA complex. (**c**) Detailed visualization of the AComA complex, including bilateral A1 and A2 segments, the fenestrated AComA, and the aneurysm, after dissection of the Heubner artery from the aneurysm dome. (**d**) Comprehensive view of the AComA complex shows the bilateral A2 segments, the left A1 segment, the fenestrated AComA, and the aneurysm after dissection of the Heubner artery and clipping of the aneurysm.

This report presents a unique case of a multilobular aneurysm associated with a fenestrated AComA. To our knowledge, this is the first video case illustrating surgical management in the context of AComA fenestrations (Figures 1, 2; Supplementary Video S1). This case highlights the relationship between structural variations and aneurysm formation within the AComA, underscoring the importance of preoperative planning and surgical adaptability in optimizing outcomes.

## Case description

A 67-year-old female presented with persistent headaches, which lead to the incidental diagnosis of a 10 mm multilobular aneurysm in the AComA (Figure 1). A right pterional craniotomy was performed for clip obliteration. Although endovascular treatment was considered, the aneurysm's multilobular morphology and broad neck made durable embolization less feasible and carried an increased risk of recurrence. All therapeutic options, including endovascular therapy, were explained to the patient, who then elected to undergo microsurgical clipping after being informed of the risks and benefits.

After a standard pterional exposure, the Sylvian fissure was sharply dissected from distal to proximal, which separated the frontal and temporal lobes and improved brain relaxation. This maneuver minimized the need for retraction of the frontal lobe. Key structures such as the ipsilateral internal carotid artery, bilateral optic nerves, and optic chiasm were widely exposed, and the lamina terminalis was fenestrated to drain CSF to obtain further brain relaxation. A small area of the gyrus rectus was resected to expose the ipsilateral A1–A2 junction and proximal A2 segment. Dissection of the AComA complex revealed two distinct anterior communicating arteries, with the larger and posterior AComA harboring the aneurysm. The right recurrent artery of Huebner (RAH) was found adherent to the aneurysm dome and was very carefully dissected free (Figure 2; Supplementary Video S1).

Preemptive identification of a temporary clipping site on the ipsilateral A1 ensured adequate proximal vascular control. A temporary clip was placed on the right A1 segment to soften the aneurysm and allow safe dissection of the dome. Pre-clipping Doppler ultrasound and indocyanine green (ICG) angiography were performed to establish baseline flow dynamics. A permanent clip was then applied to the aneurysm neck, securing the posterior bleb along with the dome. The aneurysm dome was punctured to check for residual filling. A second clip was applied to ensure complete aneurysm obliteration. ICG angiography and Doppler ultrasonography confirmed complete obliteration with normal flow in the adjacent vessels.

A routine closure was performed. The patient awoke without a neurological deficit and had an uneventful postoperative course. Follow-up angiography confirmed total aneurysm obliteration.

## Discussion

The AComA complex is a link in the vascular supply to the frontal cortex, parietal cortex, cingulate cortex, corpus callosum, optic chiasm, and basal ganglia (7, 13, 22). Disruption or occlusion of the arteries in this complex can result in profound neurological deficits, including personality changes, memory impairment, and autonomic or endocrine dysfunctions. The AComA itself functions to permit collateral flow between the bilateral A1 and A2 segments, particularly in the event of occlusion or stenosis. Surgery in this region, particularly for aneurysms, requires careful treatment of the adjacent structures as well as the vessels to ensure adequate blood flow and mitigate the risk of ischemia (3, 4, 9, 23). This also requires vigilance for anatomical variations, including aplastic or hypoplastic A1 segments and fenestrations and/or AComA duplications, as these complicate the surgical approach (7, 21, 24, 25).

## Embryologic development and anatomical variability

The anatomical complexity of the AcomA arises during embryological development. Though the AComA originates as a plexiform structure, it typically coalesces into a single vessel. Structural variations, such as fenestrations and duplications, may develop when fusion fails to complete (8, 14, 15). Although Uchino et al. classified AComA duplications into three types, 1) true fenestration, 2) duplication, and 3) partial duplication, these terms are often used interchangeably due to their overlapping hemodynamic impacts on aneurysm formation (15, 20). Given the AComA's short length, distinguishing these anomalies can be challenging, with autopsy studies reporting incidences ranging from 7.5% to 40% (8, 16). For example, Serizawa et al. found anatomical variations in 60% of 27 cadaveric brains, which included plexiform structures with multiple vascular channels (33%), fenestrations (21%), and duplications (18%). Additionally, Kwak et al. found CoW anomalies in approximately 60% of patients with AComA aneurysms, underscoring the complexity and variability associated with aneurysms in this region (2). These findings shed light on the prevalence of anatomical variations as well as the importance of understanding individual anatomy in this region (13).

## Hemodynamic influences on aneurysm formation

The AComA is among the most frequent sites for saccular cerebral aneurysm formation, accounting for 30%–37% of intracranial aneurysms (4, 5). Physiologically, the AComA can adjust its size in response to changes in flow rate; however, prolonged high flow rates beyond physiological limits can increase the risk of vascular wall damage and aneurysm formation. In asymmetrical AComA configurations, increased shear stress and stagnation points at the A2-AComA junction exacerbate hemodynamic stress, further predisposing the artery to aneurysm development. Compared to other intracranial arteries, the AComA's limited capacity for vasodilation makes it particularly susceptible to these hemodynamic effects (2, 18, 19).

Experimental studies support the link between increased blood flow and aneurysm formation. In a study by Hashimoto et al., AComA aneurysms were induced in hypertensive rats through unilateral carotid artery ligation and dietary supplementation with r-aminopropionitrile, establishing a potential causal link between elevated blood flow and aneurysm development (19).

## Theoretical basis of AComA aneurysm formation

AComA aneurysms are frequently associated with unilateral A1 segment hypoplasia. When one A1 segment is sufficiently hypoplastic, both distal anterior cerebral arteries are supplied by a single internal carotid artery. This altered blood flow pattern increases the likelihood of aneurysm formation on the AComA. Typically, when the proximal anterior cerebral arteries are asymmetrical, aneurysms tend to develop on the side of the AComA receiving the larger A1 segment. Conversely, if the A1 segments are symmetrical, aneurysms are more likely to arise from the midportion of the AComA. A predilection for aneurysm formation in patients with unbalanced anterior cerebral artery (ACA) configurations emphasizes the importance of identifying such anatomical variations preoperatively (3, 4, 17, 26).

## Impact of fenestrations on aneurysm development

Fenestrations in cerebral arteries are often asymptomatic; however, when associated with aneurysms, fenestrations can challenge attempts at treatment. Certain fenestration variants, particularly those involving the AComA, are associated with an elevated risk of ischemic events and aneurysm formation, primarily due to turbulent blood flow and the absence of tunica media in the proximal and distal regions of the fenestration (15, 16, 27).

Studies indicate that fenestrations occur in approximately 5.7%–13% of AComA aneurysm cases, with their presence closely linked to hemodynamic changes that elevate aneurysm risk (14, 21). Fenestrations within the AComA are of particular clinical concern, as these structural anomalies disrupt normal hemodynamics, increasing turbulence and wall shear stress at arterial junctions, thereby contributing to aneurysm formation and an increased risk of rupture. It is crucial for surgical planning to recognize these, as well as other common anomalies, such as hypoplastic A1 segments, which may coexist in the same patient (16, 18).

Herein we detailed a case of a multilobular aneurysm associated with a fenestrated AComA. To our knowledge, this is the first video documentation of surgical management for an aneurysm within a fenestrated AComA, thereby providing insights into the technical challenges posed by this vascular anomaly (Supplementary Video S1).

## Imaging limitations and their impact on surgical challenges

AComA aneurysms are among the most complex to manage surgically due to frequent anatomical variations, diverse aneurysm orientations, and visualization challenges during surgery and angiography. In our case, computed tomography angiography (CTA) was used for preoperative evaluation, which demonstrated the multilobular aneurysm but did not reveal the fenestrated nature of the AComA. This limitation reflects the reduced sensitivity of CTA for detecting subtle vascular anomalies. Traditionally, digital subtraction angiography (DSA) has been regarded as the gold standard for aneurysm evaluation; however, its two-dimensional nature may obscure overlapping vessels and limit precise anatomical assessment. To overcome these shortcomings, three-dimensional rotational angiography (3DRA) has emerged as an advanced imaging modality that provides superior anatomical detail and facilitates more accurate surgical planning.

Recent studies suggest that 3DRA, especially with multiplanar reconstruction, provides more detailed anatomical information compared to conventional two-dimensional DSA. For example, only 1 of 12 AComA fenestrations visualized by 3DRA was detectable on conventional 2D DSA. However, the spatial resolution of 3DRA may still fail to detect ultra-thin bridging vessels within fenestrations, which can be as small as 0.1–0.3 mm (4, 11, 17, 20).

In patients with recent subarachnoid hemorrhage, vasospasm may lead to incomplete angiographic filling, limiting angiographic assessment even with advanced techniques. The hematoma itself can also obscure the vascular and neural structures surrounding the aneurysm. Surgical navigation in such cases requires adaptability and reliance on intraoperative imaging to confirm vessel integrity (4, 24).

## Surgical vs. endovascular considerations

Endovascular coiling is an established treatment for many AComA aneurysms, particularly those that are saccular, small, and have a narrow neck. However, complex morphologies such as multilobular or broad-necked aneurysms remain challenging for endovascular therapy due to the risk of incomplete occlusion, coil compaction, or parent vessel compromise. Microsurgical clipping, in contrast, allows for direct visualization of the aneurysm neck and perforators, providing a durable occlusion while preserving critical vessels (11, 16, 28). In the present case, these preoperative considerations favored clipping over embolization. The intraoperative discovery of a fenestrated AComA further confirmed the value of the microsurgical approach, as direct dissection was required to preserve the recurrent artery of Heubner and other perforators.

## Surgical techniques and intraoperative considerations

Effective surgical management of AComA aneurysms, particularly in the presence of variant anatomy, requires meticulous preoperative planning and precise operative technique. In the present case, we used the standard pterional and transsylvian approach which provided optimal aneurysm exposure while minimizing brain retraction. When the aneurysm sac obscures fenestrations, surgical exposure and clipping become increasingly difficult. The often thin and delicate bridging arteries within these anomalies supply vital structures including the optic chiasm, hypothalamus, and subcallosal region. Thus, any damage to these vessels can lead to severe complications such as memory impairment, visual deficits, and endocrine disturbances (4, 7, 22).

Initial dissection requires avoiding direct contact with the aneurysm dome and focusing on exposing the parent artery and aneurysm neck. Proper clip selection and placement are essential to maintain blood flow to perforating vessels. Complete dissection of the “H” complex, formed by the bilateral A1 and A2 segments and the AComA, and separating perforating vessels from the aneurysm neck is necessary for a favorable outcome, especially when the aneurysm lies behind the parent vessel (4, 6). In the present case, the RAH was closely adherent to the aneurysm dome, posing additional risk; its preservation was crucial given its role in supplying the basal ganglia and hypothalamus (Figure 2; Supplementary Video S1).

## Anatomical preservation and surgical adaptability

Understanding the detailed vascular anatomy is fundamental to successful AComA surgery. The AComA perforators, categorized by Türe et al. into hypothalamic, subcallosal, and median callosal arteries, supply critical brain regions. Damage to these vessels can result in profound neurological sequelae including cognitive and personality changes. Anastomoses between perforators, particularly in the hypothalamic branches, necessitate careful handling during surgery. During aneurysm intervention, maintaining the patency of all AComA channels is recommended, especially with fenestrated AComAs, as occluding any of these can disrupt blood flow to downstream A1 and A2 segments. Surgical complications, such as vasospasm or inadvertent injury, affecting these perforators can lead to poor outcomes (3).

Intraoperative tools, including Doppler ultrasound and ICG angiography, are useful in assessing flow dynamics and confirming vessel patency before and after clipping (3, 4). Temporary clipping of the A1 segment before definitive aneurysm clipping, as employed in this case, allows for controlled dissection and minimal rupture risk. This case highlights the value of an adaptive surgical strategy that accounts for unexpected anatomical variations within the AComA complex.

## Conclusion

The successful treatment herein of a complex, multilobular aneurysm within a fenestrated AComA demonstrates the importance of recognizing anatomical variations and tailoring surgical strategy accordingly. Detailed preoperative imaging, meticulous intraoperative dissection to preserve critical vessels, and use of intraoperative tools to guide decision-making are crucial factors in achieving a favorable outcome. This case highlights the necessity of an adaptive surgical approach when managing AComA aneurysms complicated by other vascular anomalies. Future studies should explore advanced imaging technologies and refined surgical techniques to improve outcomes in these challenging cases.

## Data Availability

The original contributions presented in the study are included in the article/Supplementary Material, further inquiries can be directed to the corresponding author.

## Supplementary material

Supplementary Video S1 Intraoperative video demonstrating AComA fenestration recognition, perforator preservation (RAH), and clip application.
